# Single-cell analysis of VACV infection reveals pathogen-driven timing of early and late phases and host-limited dynamics of virus production

**DOI:** 10.1371/journal.ppat.1012423

**Published:** 2024-08-02

**Authors:** Liam Michael Howell, Nicholas Peter Gracie, Timothy Peter Newsome

**Affiliations:** 1 School of Life and Environmental Sciences, The University of Sydney, Sydney, Australia; 2 Sydney Institute for Infectious Diseases, The University of Sydney, Sydney, Australia; Thomas Jefferson University, UNITED STATES OF AMERICA

## Abstract

The extent and origin of variation in the replication dynamics of complex DNA viruses is not well-defined. Here, we investigate the vaccinia virus (VACV) infection cycle at the single-cell level, quantifying the temporal dynamics of early and post(dna)-replicative phase gene expression across thousands of infections. We found that viral factors determine the initiation time of these phases, and this is influenced by the multiplicity of infection (MOI). In contrast, virus production dynamics are largely constrained by the host cell. Additionally, between-cell variability in infection start time and virus production rate were strongly influenced by MOI, providing evidence for cooperativity between infecting virions. Blocking programmed cell death by pan-caspase inhibition increased infection frequency but not virus production at the population level due to a concurrent attenuation of per-cell virus yield, suggesting a dual role for caspase signaling in VACV infection. Our findings provide key insights into the pivotal factors influencing heterogeneity in the infection cycle of a large DNA virus at the single-cell level.

## Author summary

The successful transmission and progression of a viral infection depends on the initial infectious virus dose, which in turn is defined by the efficiency of virus replication within host cells. Understanding what determines the productivity of individual cells, and the variability between them, is a central question in the emerging field of single-cell virology.

Our study investigates the single-cell infection dynamics of vaccinia virus (VACV), a large, complex, DNA virus, revealing a profound influence of initial infectious dose on the timing and rate of progression of virus replication. Cells infected with a low dose take over three times longer to initiate virus production compared to those with a higher dose. However, the dynamics of the second phase of infection, de novo production of virus, are unperturbed by this variable: a higher infectious dose does not equate to a greater yield. Furthermore, we observed that the proportion of cells advancing to productive replication increases markedly with the virus dose. This effect is compounded by the suppression of apoptosis, a cell death process, suggesting that induction of apoptosis is effective in pruning the population of susceptible host cells. This has broad implications for the pathogenesis of viral diseases as the infectious dose will govern the window of opportunity for the host immune system to effectively respond to the infection. This research contributes to the broader field of single-cell virology by highlighting the complex relationship between virus dose and infection dynamics at the cellular level.

## Introduction

Infectious dose and rates of viral shedding are key factors influencing disease transmission patterns. These variables are inherently a product of virus replication dynamics. To date, our understanding of virus replication has largely been shaped by population-level assays, which do not account for the intrinsic heterogeneity present at the level of single cells. The field of single-cell virology encapsulates genomics, transcriptomics and imaging approaches and has revealed profound between-cell variability in the infection kinetics of many viruses [1–3]. Heterogeneity in the initiation of infection, replication speed, and the length of the replication phase is characteristic of poliovirus (PV)-infected cells, as revealed by live-cell imaging of single cells isolated in microfluidic chambers [4]. Multiplicity of infection (MOI, the ratio of infectious virus per cell) was found to play a profound role in influencing the dynamics of aspects of the PV infection cycle, consistent with observations identifying infectious dose as a major determinant of disease outcomes at the organismal level (Influenza A virus (IAV): [5,6], and SARS-CoV-2: [7,8]).

Most single-cell virology studies to date have concentrated on RNA viruses. The complex interplay between host and viral determinants that shape the single-cell infection trajectory of DNA viruses—which in contrast to RNA viruses have relatively low mutation rates [9–11]—remains largely unexplored. Large DNA viruses are further distinguished from RNA viruses by their complex infection cycles in which sets of genes are expressed in tightly regulated stages, including early and post(DNA)-replicative (PR) phases at minimum. Functions of early genes typically encompass immunosuppressive and anti-apoptotic activities; critical in establishing an environment conducive to productive infection [12]. PR genes generally encode proteins involved in DNA binding and packaging, transcription factors, mediators of virion maturation, and structural proteins [13–15]. Mechanisms underlying the variability of the establishing (early) and the productive (PR) phases at the single-cell level have not been described.

The prototypal poxvirus vaccinia virus (VACV) offers a valuable platform to investigate single-cell variability during infection with a DNA virus, due to our deep understanding of its biology and the wealth of genetic tools available for experimental manipulation. VACV strains incorporating multiple phase-specific reporters have been previously published [16,17], and have proven to be valuable tools in understanding the dynamics of, and relationships between, these phases. We therefore engineered a VACV incorporating two fluorescent proteins, allowing us to assay the early and PR phases of infection with live-cell microscopy. We were able to detect the initiation of infection as early as 40 minutes, and identify stalled, productive, and lytic events. By integrating machine learning for image segmentation, we optimized our data collection pipeline, capturing the dynamics of individual infections with high temporal resolution at scale.

Our findings reveal high variability in functionally relevant outcomes of VACV infection at the level of single cells. We also demonstrate that the timing of both early and PR gene expression is strongly influenced by MOI, and that the degree of between-cell variability in the timing of early gene expression negatively correlates with MOI. Interestingly, increasing infectious dose leads to a concurrent increase in cell-to-cell heterogeneity in the rate of virus production, but the dynamics of virus production are otherwise insensitive to this variable. Disrupting cellular defenses by treating VACV-infected cells with the pan-caspase inhibitor Q-VD-Oph efficiently blocked cell death and increased infection frequency but was associated with a concurrent reduction in per-cell virus yield. Caspase signaling, therefore, exerts both proviral and anti-viral effects at different stages of the replication cycle.

## Results

### Variability of VACV infections at the single-cell level

**Fig 1:**
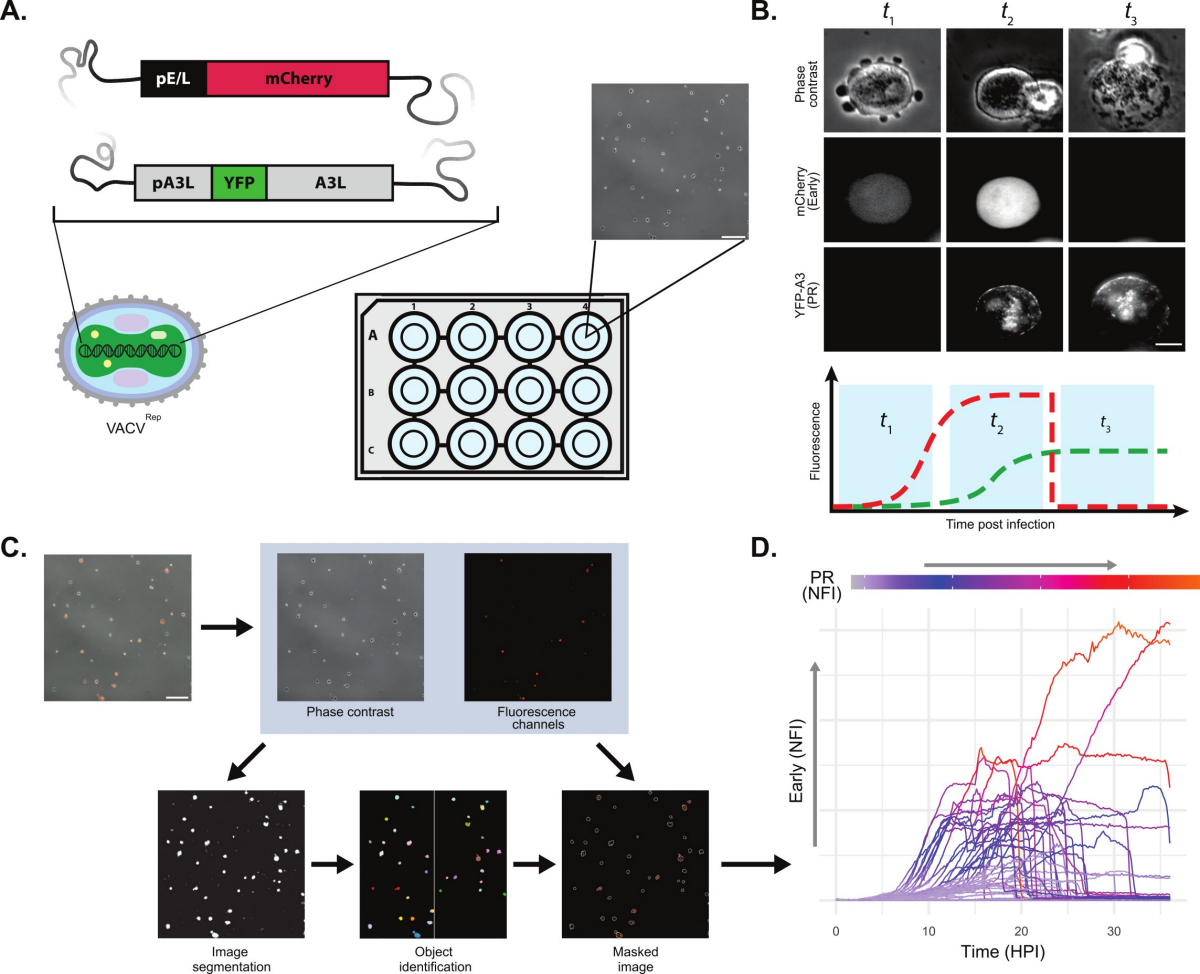
High-throughput single-cell analysis of the VACV infection cycle. **A**, **B**, HeLa cells, cultured in 12-well plates at low density, were infected with Vaccinia virus (VACV) expressing two fluorescence reporters regulated by Early (pE/L) and PR (pA3L) promoters, enabling monitoring of early infection events, replication, and lysis. **C**, Widefield fluorescence microscopy images were segmented by a machine learning classifier using phase contrast channel data, generating masks which were applied to fluorescence channel images. **D**, Quantification of normalized fluorescence intensity (NFI) per cell and temporal tracking of single-cell infection events (n = 50 cells randomly sampled). Scale bars: 200 μm (**A**, **C**); 10 μm (**B**).

To monitor VACV infection stages in real-time, we recombined viruses bearing the fluorescent markers pE/L-mCherry (Early, [18]) and pA3L-YFP-A3 (PR, [13,19]) to generate a reporter virus we termed VACV^Rep^ (Fig 1A and S1 Video). Imaging VACV^Rep^ enabled us to rapidly detect the establishment of infection (by mCherry fluorescence) and observe the timing and dynamics of the production of new virus particles (by YFP-A3 fluorescence). Cell lysis can be observed by the rapid loss of cytoplasmic mCherry that results from the disruption of cell membrane integrity (Fig 1B). To confirm that YFP-A3 fluorescence was an appropriate proxy for infectious virus production, we infected duplicate cell populations at an MOI of 5 and assayed both fluorescence intensity and infectious progeny production (by lysis and titring by plaque assay) over 24 hours. We observed a strong correlation between these parameters (R^2^ = 0.98, S1A and S1B Fig). As expected, the addition of the DNA replication inhibitor arabinoside cytosine (araC) led to a striking reduction of YFP-A3 fluorescence and infectious virus production (S1C Fig). The earliest time point at which we detected mCherry fluorescence was 40 minutes, lagging approximately 20 minutes behind previously reported earliest detections of early viral mRNA [20]. Although this lag is consistent with the requirement for mRNA translation, fluorophore maturation, and the accumulation of sufficient fluorescence signal to pass the limits of detection in our system, our reported start times for Early and PR expression should be considered in the context of this intrinsic delay.

Combining time-lapse microscopy of VACV^Rep^ infected cells with image segmentation leveraged by machine learning enabled us to capture detailed information on the infection cycle in individual cells at scale (Fig 1C and 1D). For the purposes of this study, we define the ’infection cycle’ as the period from virus addition to the end of productive replication, and reserve ’replication cycle’ for the period of PR gene expression and virus assembly.

**Fig 2:**
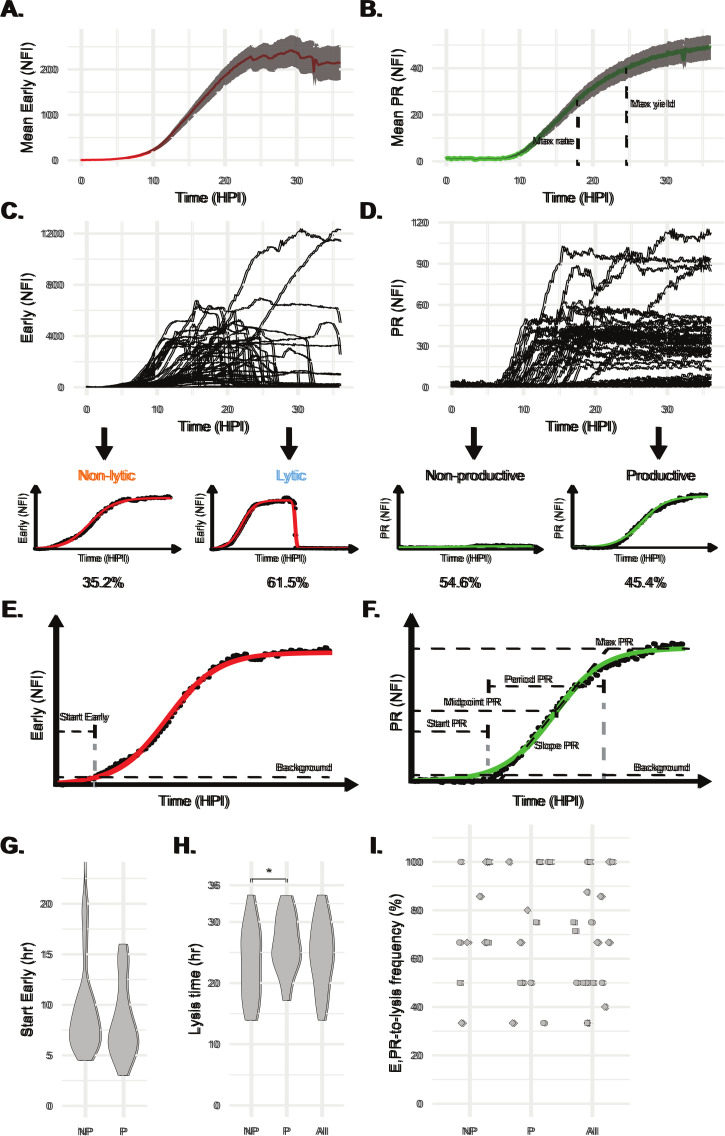
Between-cell variability in VACV infection dynamics and outcomes. **A**, **B**, HeLa cells were infected with VACV^Rep^ at an MOI of 1 and the normalized fluorescence intensity (NFI) of individual cells over time was quantified (n = 91). Means calculated from all infections analyzed indicated a typical progression of VACV infection. **C**, **D**, The underlying temporal dynamics at the single cell level revealed substantial between-cell variability (n = 50 randomly selected cells). Sigmoidal or double-sigmoidal models were fitted to single-cell fluorescence data, categorizing infections as non-lytic or lytic (based on Early NFI), and productive or non-productive (based on PR NFI). Percentages beneath each model represent the proportion of cells in that category. **E**, **F**, Key parameters describing single-cell infection dynamics were extracted from the fitted models. **G**, Comparison of infection start time between non-productive (NP) and productive (P) infections. **H**, Comparison of the timing of lysis between P, NP, and All infections. **I**, Comparison of the frequency of lysis between P, NP, and All infections. Points represent the mean of a field of view, while shapes represent replicate wells. Statistical comparisons were performed using unpaired Student’s T-tests. Comparisons with no annotation of significance shown were not significantly different. Error bars represent SEM (**A**, **B**).

To investigate the between-cell variability in VACV infection dynamics we infected cells at an MOI of 1, reasoning that a lower MOI would be more likely to represent a balance point between host defenses and viral antagonism. At this MOI, 85% of VACV^Rep^-exposed cells initiated Early expression, with a median start time of 7.67 hours post-infection (HPI). Productive infections at MOI 1 were 42.5% of total cells (50% of infected cells), less than the 63.2% predicted by a Poisson distribution. This might reflect variable infection efficiency due to cell type and confluency (PFU was calculated in confluent BS-C-1 monolayers). Although 57.5% of infected cells failed to progress to productive replication, non-productive infections did not start significantly later than productive infections (Fig 2G). We conclude that a proportion of infections are substantially stalled at the early gene expression phase.

Within 36 HPI, 50.0% of infected cells progressed to productive replication. On average the rate of virus production (rate of increase of PR fluorescence) reached a maximum at 17.7 HPI, while the total yield of virus (maximum PR fluorescence) plateaued at 24.8 HPI (Fig 2A and 2B). Sigmoidal models ([4], see Methods) fitted 98.6% of observed infections and enabled the extraction of metrics describing key features of single-cell viral infection kinetics (S1 Table and Fig 2C and 2D). Given that VACV^Rep^ expresses mCherry from a dual Early/Late promoter, the activities of which cannot be separated in our analysis, we largely restricted our examination of Early NFI to determining the onset of viral gene expression (Start Early, Fig 2E). From PR NFI, we defined the onset of productive replication (Start PR), the rate of virus production (Slope PR), the time taken from start of imaging to reach half of maximum virus production (Midpoint PR), the time between initiation of replication and maximum yield (Period PR), and total virus yield (Max PR, Fig 2F). The delay between infection and the initiation of replication was extrapolated from Start Early and Start PR values (E-to-L Delay). We found that all of these parameters varied dramatically across the infected cell population, and that this variability was not substantially explained by between-cell morphological variation detectible in our dataset (S2 Fig).

Infections were fitted by either sigmoidal or double sigmoidal models based on Early NFI and were classified as non-lytic or lytic. At MOI 1, 61.5% of cells lysed by 36 HPI (35.2% non-lytic, 3.3% unclassifiable, n = 91). New lytic events mainly occurred at 15–32 HPI (95%), with only 2.8% after 32 HPI. Non-productive infections lysed earlier (p = 0.017, Fig 2H), but had similar lysis frequencies to productive infections (Fig 2I). Collectively these data demonstrate that the timing of lysis is highly variable, a relatively large fraction of infected cells reach their maximum virus yield and fail to lyse over an extended period, and progression to productive replication is associated with late lysis.

### Correlations of single-cell infection parameters

**Fig 3:**
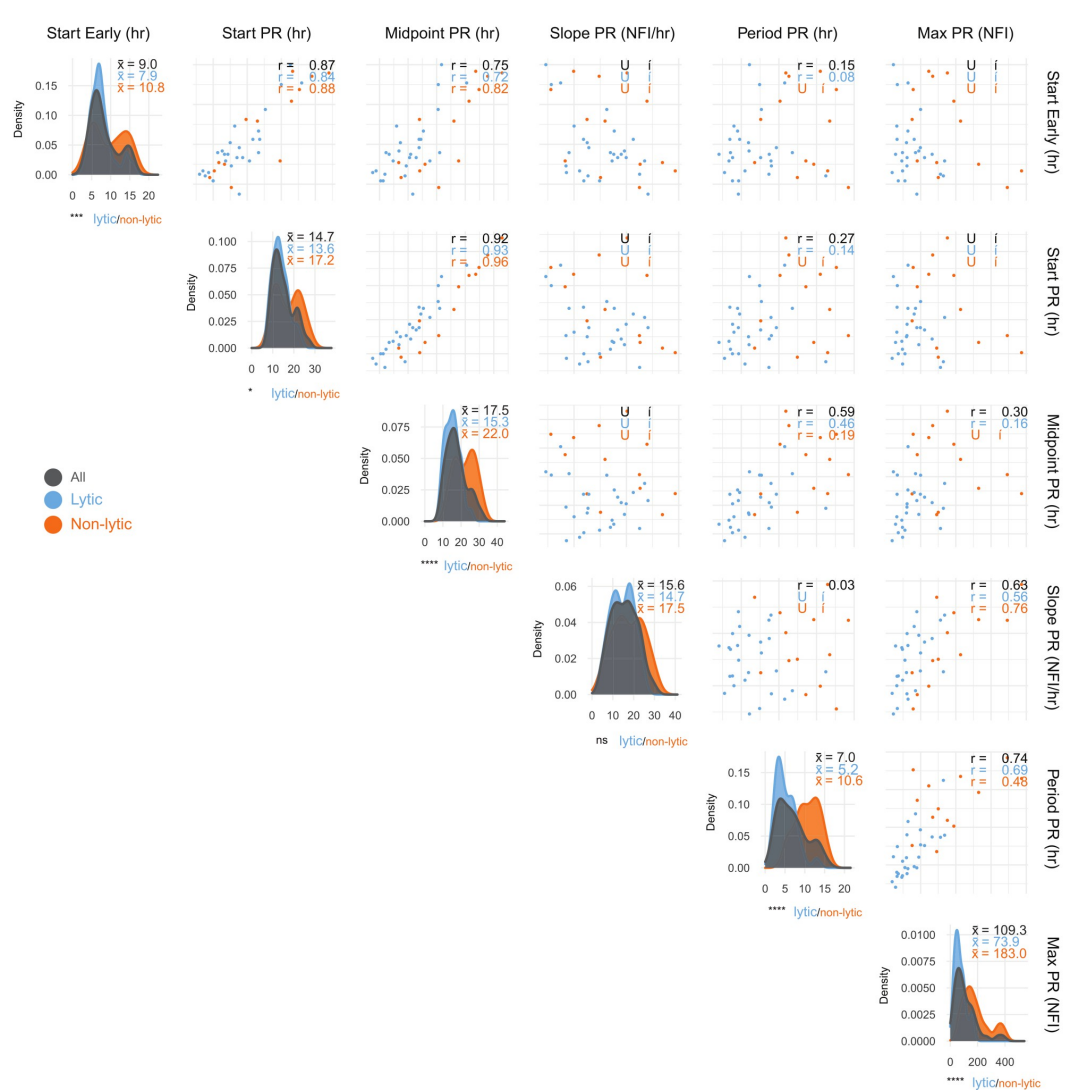
Correlations of key parameters describing VACV infections. Parameters extracted from models fitted to VACV^Rep^ infections at an MOI of 1 were evaluated via pairwise correlations (Pearson’s correlation coefficient, upper triangle) and density distributions ($\bar{x}$ = mean, diagonal) for total (n = 40), lytic (n = 27), and non-lytic (n = 13) cell populations. Density indicates the relative frequency of values within the dataset. Parameters demonstrating strong correlation likely share an underlying mechanism or reflect the same facet of the host-virus interface. Statistical comparisons were performed using unpaired Student’s T-tests.

To examine the relationships between single-cell infection parameters we calculated pairwise correlations, enabling insights into shared mechanisms driving distinct aspects of the infection cycle. Correlations between Midpoint PR and both Start Early and Start PR (R = 0.75, 0.92, Fig 3) suggest a common mechanism, consistent in lytic (R = 0.72, 0.93) and non-lytic (R = 0.82, 0.96) infections. Start Early and Start PR showed no correlation with Max PR or Slope PR, indicating initiation time does not predict yield (-0.16<R<-0.05). However, Slope PR and Max PR were positively correlated (R = 0.63–0.76); faster replication generally yields more virus. Start Early and Start PR also correlated strongly in both classes (R = 0.84–0.88).

Significant differences were observed between lytic and non-lytic infections in most parameters (Fig 3). These differences remained even when model fitting and analysis were performed on a dataset limited to 24 HPI, suggesting that these two classes display divergent characteristics regardless of when lysis occurs (S3 Fig). The two classes differed significantly in both initiation of viral gene expression (Start Early; $\bar{x}$ = 7.9 and $\bar{x}$ = 10.8, p<0.001) and rate of progression to productive replication (E-to-L Delay; 6.0 vs 7.2 hrs, p<0.05). While both replicated at similar rates (Slope PR), non-lytic infections had a longer replication period (Period PR; $\bar{x}$ = 10.6 vs $\bar{x}$ = 5.2) and more than double the yield (Max PR; $\bar{x}$ = 183.0 vs $\bar{x}$ = 73.9). There is an association between lower virus production and propensity for lysis at the cellular level, either because lysis acts as a cellular mechanism to curb virus yield or because lysis occurs as a result of exhaustion of cellular resources.

### Contributions of viral and host factors to infection outcomes

Modulating the amount of virus delivered to cells has been observed to impact infection dynamics at the single-cell level [4], as well as being associated with faster disease onset and worsened clinical outcomes at the organismal level [21,22]. We investigated the impact of varying the MOI of VACV^Rep^ infections on single-cell infection dynamics, providing insight into the relative contributions of viral and host factors in regulating the limits of infection cycle parameters.

**Fig 4:**
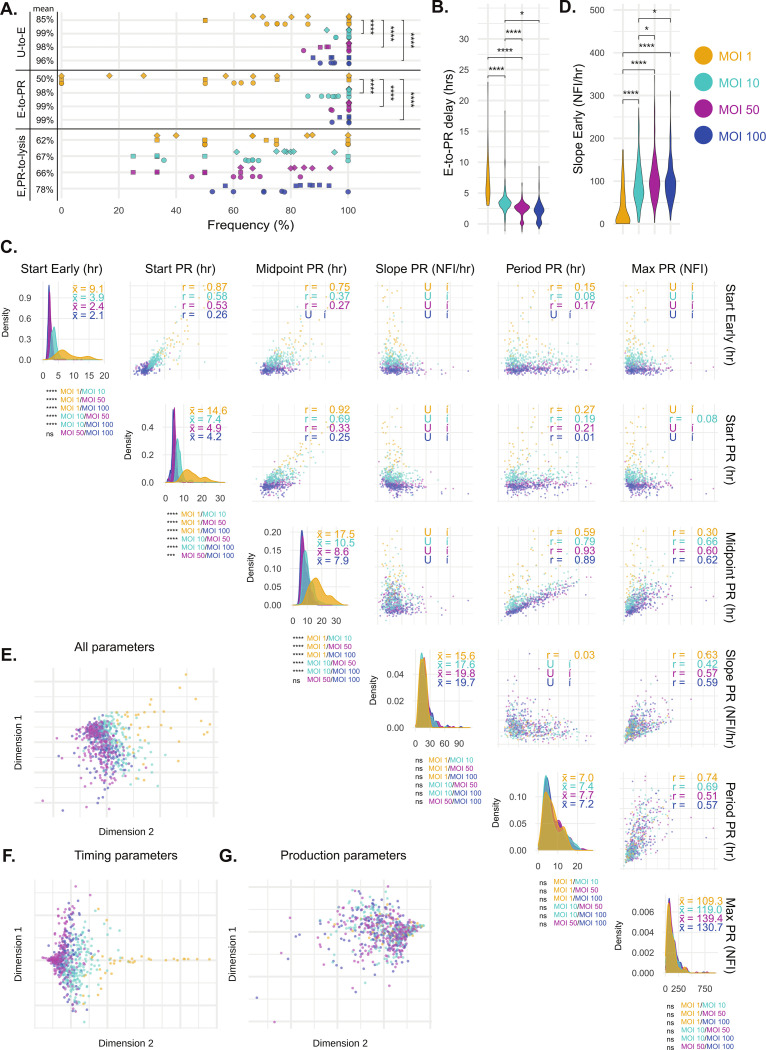
Modulation of VACV infection outcomes by varying MOI. A, Frequencies of infection (U-to-E; uninfected to Early gene expression), progression to productive replication (E-to-L; Early gene expression to PR gene expression), and lysis (E, L-to-lysis) for cells exposed to VACV^Rep^ at MOIs of 1, 10, 50 and 100. Points indicate the average of a field of view, while point shapes indicate replicate wells. B, Distributions of temporal delay values between initiation of Early and PR reporter expression for individual infections at MOI 1, 10, 50 and 100. C, Parameters extracted from models fitted to single-cell fluorescence data (MOI 1; n = 88 cells, MOI 10; n = 232 cells, MOI 50; n = 205 cells, MOI 100; n = 200 cells) were analyzed via pairwise correlations (Pearson’s correlation coefficient, upper triangle) and density distributions ($\bar{x}$ = mean, diagonal). Density indicates the relative frequency of values within the dataset. D, Rate of Early NFI accumulation at MOI 1, 10, 50 and 100. E, F, G, Two groups of parameters separated by a stronger (Timing parameters: Start Early, Start PR, and Midpoint PR) or weaker (Production parameters: Slope PR, Period PR, Max PR) response to change in MOI were compared by Multidimensional Scaling. Statistical comparisons were performed using Student’s T-tests (B, C, D), or chi-squared tests (A). Comparisons with no annotation of significance shown were not significantly different.

Increasing MOI above 1 led to significantly more Early-expressing and PR-expressing cells but both parameters appeared to reach an upper limit of susceptible cells at MOIs above 10 (Fig 4A). Cells that remained uninfected were a combination of live cells resistant to infection and cells that underwent cell death prior to early gene expression (approximately 20% live uninfected cells at final time point at MOI 1, less than 1% at MOI 10, 0% at MOI 50 and MOI 100). Progression from viral gene expression initiation to productive replication (E-to-L delay) accelerated with higher MOI across all comparisons (Fig 4B). At high MOIs, infections initiated earlier (Start Early) and progressed to PR phase (Start PR) much more rapidly. A 10-fold increase in MOI, from 1 to 10, accelerated these phases sufficiently for 95% of infections at MOI 10 to begin before 19% of infections at MOI 1. However, the correlation between Start Early and Start PR weakened dramatically with increasing MOI (R = 0.87–0.31 at MOI 1–100), suggesting a bottleneck between these two events that higher virus dose cannot readily overcome.

MOI did not significantly impact virus production per cell (Max PR), duration of productive period (Period PR), or virus production rate (Slope PR, Fig 4C). However, early gene expression rate (Slope Early) did increase significantly with MOI, even in non-productive infections (Fig 4D). From these observations, we were able to delineate two parameter categories: those sensitive to MOI (Timing: Start Early, Start PR, Midpoint PR) and those largely limited by the host (Production: Slope PR, Period PR, Max PR). Timing parameters relate to the onset of Early and PR expression, while Production parameters describe the dynamics of progeny virus production. The time taken to reach half of maximum virus production (Midpoint PR) supports the distinction between these two categories as this parameter is a function of both the timing of infection (virus limited) and the rate of virus production (host limited). Increasing MOI decouples the correlation of Midpoint PR with Start Early (Midpoint PR with Start Early: R = 0.75–0.14 at MOI 1–100) and Start PR (Midpoint PR with Start PR: R = 0.92–0.29 at MOI 1–100). One parameter within the pairing readily shifts in response to MOI, while the other does not.

Multidimensional scaling of all single-cell infection parameters (MDS) showed clear clustering by MOI that was greatly enhanced when considering only Timing parameters (Fig 4E and 4F). Clustering was absent when considering only Production parameters (Fig 4G). Variance explained by MOI was 61-fold higher in Timing than in Production parameters (PERMANOVA; R^2^ = 0.62 vs 0.01), consistent with initial infectious dose being the main driver of these parameters. Interestingly, increasing MOI also correlated with reduced between-cell variability in the timing of the onset of early phase (Start Early) and increased between-cell variability in the rate of virus production (Slope PR), but did not otherwise notably impact variability in the dynamics of PR expression (S2 Table). This suggests that while MOI impacts the timing of infection events, host factors are the main limiter of production dynamics.

### The impact of cell death inhibition on VACV infection dynamics

Viruses both induce and inhibit cell death at various stages of their replication cycles, primarily through the manipulation of cell death signaling pathways such as apoptosis, necroptosis, and pyroptosis [23]. Typically, viruses inhibit programmed cell death in the early stages of infection to ensure the host cell remains viable for productive replication [24,25]. As the infection progresses, many viruses including IAV, PV and Respiratory Syncytial Virus (RSV) activate cell death pathways for optimal virus production, release, and spread [26–28]. Although cell death in the VACV infection cycle has been observed late (12–36 HPI) and demonstrated to occur concurrently with caspase activation [29,30], its role remains undefined.

**Fig 5:**
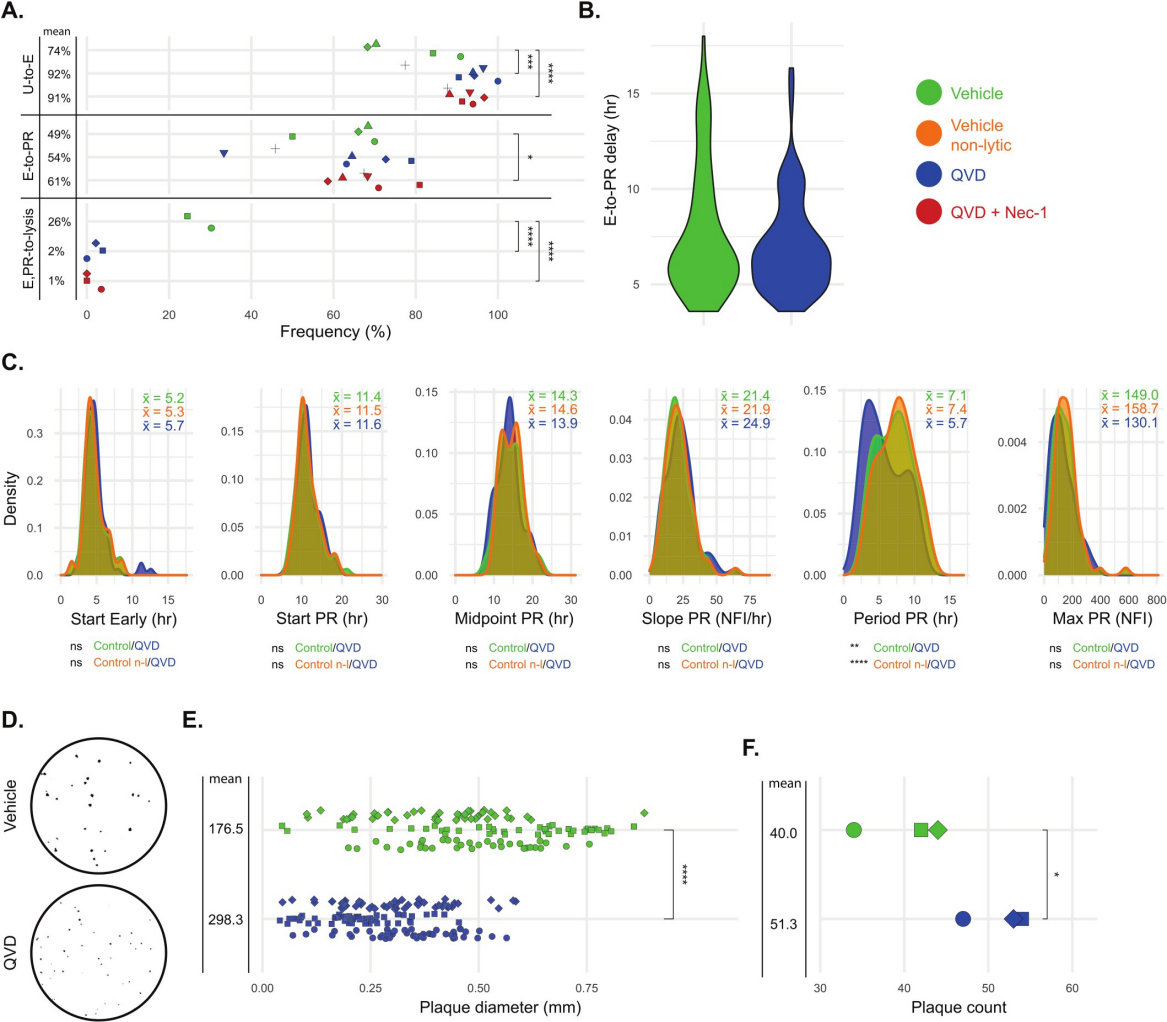
Impact of inhibition of programmed cell death on VACV infection dynamics. HeLa cells were infected with VACV^Rep^ at an MOI of 5, with or without 50 μM Q-VD-Oph (QVD), a pan-caspase inhibitor, and with and without 40 μM Necrostatin-1, a RIPK1 inhibitor. **A**, Frequencies of infection (U-to-E), progression to productive replication (E-to-L), and lysis (E, L-to-lysis). Points indicate the average of a field of view, while point shapes indicate replicate wells. **B**, Distributions of temporal delay values between initiation of Early and PR reporter expression for individual infections in the presence and absence of QVD. **C,** Density distributions of single-cell infection parameters were compared between QVD (n = 81 cells) and all vehicle only infections (n = 76 cells), as well as QVD and vehicle non-lytic infections only (n = 66 cells). **D**, **E**, **F**, Plaque assay performed on HeLa cells treated with QVD (50 μM) versus a vehicle control. One representative well is shown (**D**). Statistical comparisons were performed using unpaired Student’s T-tests (**B**, **C**) or chi-squared tests (**A**, **E**, **F**). Comparisons without significance annotation shown were not significantly different.

We have previously observed that at MOI 1 a proportion of VACV^Rep^-infected cells stall at Early stage (54.6%, Fig 2D), and most of these cells undergo programmed cell death (60%, S3 Table), indicating that programmed cell death is acting as a defense against VACV replication. To determine the impact of cell death on virus production, we examined the effect of inhibiting caspase-dependent programmed cell death with the irreversible pan-caspase inhibitor QV-D-Oph (QVD). An MOI of 5 was used for the following experiments, which represented a compromise between a high frequency of productively infected cells and maintaining a competitive interplay between host and viral elements. QVD treatment of cells infected by VACV^Rep^ at MOI 5 was effective at blocking cell lysis, implicating a caspase-dependent mechanism (Fig 5A).

Treatment with QVD resulted in an increase in the proportion of cells exposed to virus that became infected (Early expression) but did not alter how frequently those infected cells successfully progressed to productive replication (PR expression), leading to an overall increase in the number of productive infections at the population level. Blocking necroptosis by simultaneous treatment with the RIPK1 inhibitor necrostatin-1 did not increase cell survival more than QVD alone but did result in a small increase in the frequency of progression to productive replication. It is feasible that the increased number of infected cells in the QVD-treated condition is drawn from a pool of cells that would otherwise undergo programmed cell death. Supporting this, cell lysis of uninfected cells (non-Early-expressing) was dramatically curtailed following QVD treatment (S4 Table). Having observed that blocking programmed cell death led to a greater number of productive infections in an exposed cell population, we examined single-cell infection parameters under caspase inhibition.

We observed that QVD treatment did not significantly alter the rate of progression from Early to PR stages (Fig 5B) or the pairwise correlations between infection parameters (S4 Fig). There was little effect on the distributions of most infection parameters except for infection period, which was truncated in QVD-treated cells compared to control cells (Period PR; $\bar{x}$ = 5.7 versus $\bar{x}$ = 7.1, Fig 5C). This led to a 13% reduction in the productivity of infected cells (Max PR; $\bar{x}$ = 130.1 versus $\bar{x}$ = 149.0) that did not reach significance (p = 0.15). QVD treatment is effective in preventing cell death before Early gene expression, but also reduces the amount of virus each productively infected cell can produce. Based on this single-cell data we predicted that these opposing effects would largely counteract each other at the population level (Max PR Δ$\bar{x}$ = 18.9 per productive cell; Δ$\bar{x}$ = 2.2 adjusted for all infected and uninfected cells). This prediction was borne out by one-step growth assay, which revealed no significant difference in viral replication kinetics in the presence or absence of QVD (S5 Fig). Viral plaques are initiated by a single infected cell and, as such, their formation has been proposed as a proxy for elucidating aspects of the dynamics of single cell infections [1,31]. Strikingly, a plaque assay recapitulates our observation of single cell dynamics under caspase inhibition. VACV^Rep^ plaques were more numerous (40.0±5.3 versus 51.3±3.8, p = 0.044) but reduced in size (0.48±0.18 mm versus 0.29±0.12 mm, p < 2.2 x e^-16^) when QVD was present in the overlay (Fig 5D–5F). Thus, cell death acts to prune the population of cells susceptible to productive infection.

## Discussion

Using a dual reporter virus, we tracked key events in the VACV infection cycle at the cellular level, including the onset of early gene expression, the onset and dynamics of productive replication, and cell lysis. Our approach was enabled by machine-learning identification of cells and automated cell tracking, with high reproducibility and robustness. This is the first large-scale study of the single-cell infection dynamics of a large, complex DNA virus.

Our data reveal substantial variability across an infected cell population in all measured parameters. At low MOI, a proportion of Early-expressing cells failed to progress to productive replication (50%, Fig 4A), indicating that genome replication may be an important bottleneck that is sensitive to the initial stoichiometry of infection. Assaying genome replication in live single cells is technically challenging. For example, genome replication of VACV is potently inhibited by dsDNA binding dyes previously used in live cell imaging [32]. Future studies, incorporating an intermediate-specific promoter, for example, could distinguish whether late phase replication is constrained before or after the initial round of DNA replication [33,34]. A triple-reporter virus that assays early, intermediate, and late phases, has been previously described and would be suited to this approach [16]. Our findings also set the foundation for incorporating loss-of-function virus mutants into our analysis pipeline; for example, to study the role of viral inhibition of apoptosis in infection outcomes.

**Fig 6:**
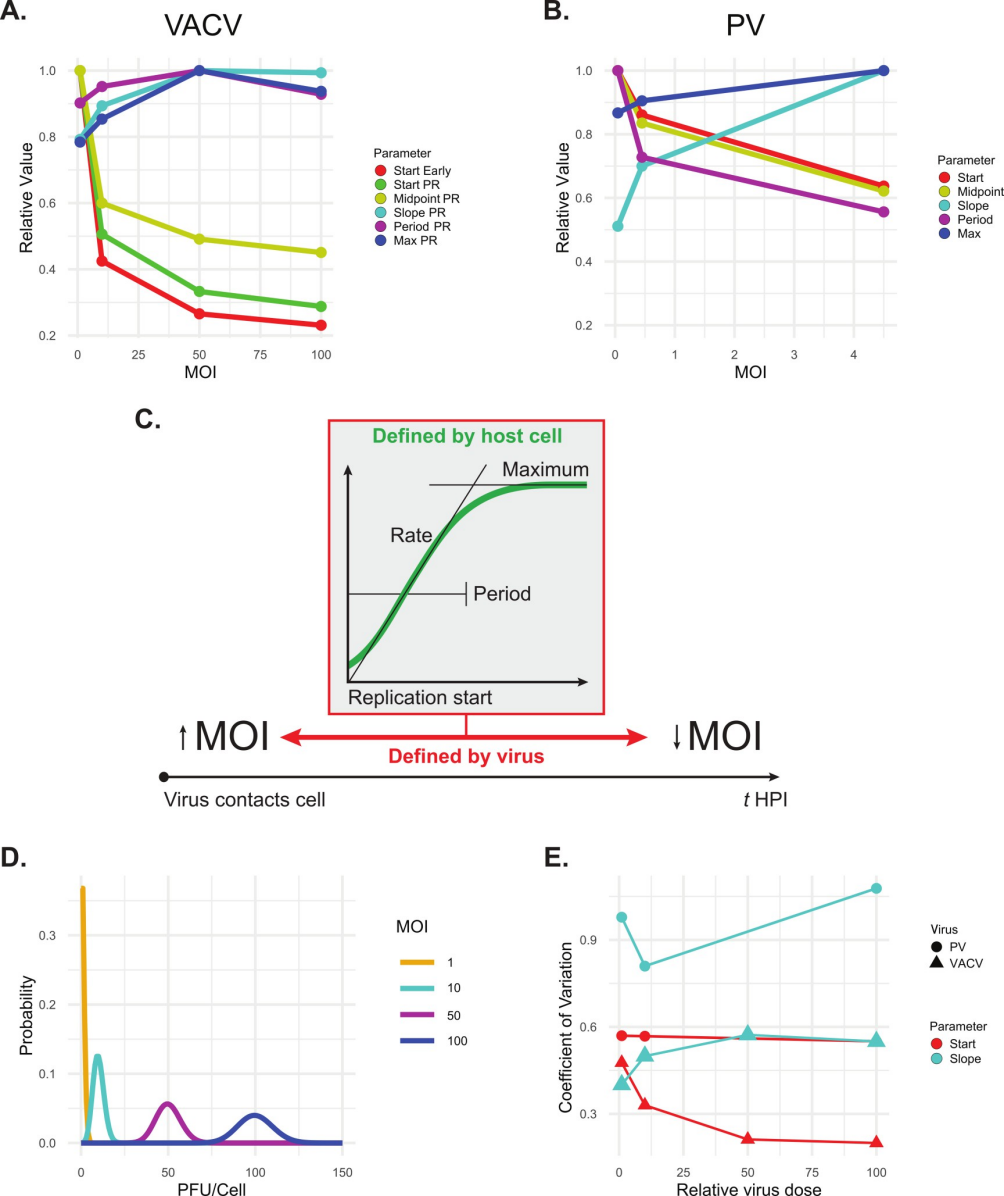
Viral versus host driven infection dynamics. **A**, **B**, VACV and PV single cell infection parameters rescaled by maximum normalization at a range of MOIs. Data first shown in Fig 4C. **C**, In our model, we propose that viral factors primarily dictate the timing of the early and PR VACV infection phases. Once the PR phase is initiated, the dynamics of virus production and assembly are limited by the host cell and are insensitive to the initial stoichiometry of infection. **D**, Poisson distributions showing the distribution of the number of infectious units (PFU) predicted to initiate an infection at the indicated MOIs. **E**, Coefficients of variation of selected single cell infection dynamics parameters for VACV and PV. Data first shown in Fig 4C. ‘Start’ for VACV represents the parameter Start Early. PV data was sourced from [4].

We observed that the timing of the onset of both the early and PR phases of the viral infection cycle were strongly influenced by modulating MOI. However, the dynamics of PR gene expression remained largely insensitive to the initial infectious dose (Fig 6A). Based on these findings, we categorized VACV single-cell infection parameters into two distinct groups: Timing (Start Early, Start PR, Midpoint PR), and Production (Slope PR, Period PR, Max PR). Our observations contrast with those from a large-scale analysis of PV infection dynamics. This analysis quantified the expression of a GFP transgene incorporated into the PV genome in single cells isolated within a microfluidics device [4]. PV is a positive-sense, single-stranded RNA virus that does not exhibit early and PR phases of gene expression. Instead, the entire PV genome functions as an mRNA, with a single open reading frame (ORF) encoding a polyprotein that is subsequently cleaved to produce all PV proteins. Compared to our results, PV start point represented our parameters Start Early and Start PR. That study found that the start times and midpoints of PV infection decreased as MOI increased (Fig 6B). Additionally, production-related parameters in PV infections showed greater plasticity than those of VACV, with the slope increasing and the replication period decreasing with increasing MOI. This suggests that increasing the initial infectious dose was generally associated with an acceleration of the PV replication cycle, with virus being produced more quickly and the initiation of lysis occurring more rapidly. In contrast, we observed that VACV exhibited a dramatically different interaction with MOI. There was no strong relationship between this variable and the replication period, the rate of replication, or the frequency of lysis. Increasing MOI was not associated with an increase in the per cell yield of progeny virus for either VACV or PV. These results suggest a model for VACV infection where PR gene expression dynamics are broadly insensitive to changes in MOI, but the timing of VACV gene expression phases is highly sensitive to it. Therefore, altering MOI causes significant changes in when early and PR gene expression takes place, but the dynamics are largely constrained by the host cell (Fig 6C).

Cell-intrinsic variability is a product of cell cycle stage, metabolic history, arrangement of organelles, microenvironmental variation (including cell stress), and epigenetics, among many other factors [1]. Cell populations, even when cultivated in controlled environments, exhibit desynchronized cell cycles due to stochastic variations in gene expression [35,36]. These variations influence the availability of resources necessary for viral replication [37–39]. However, not all viruses display replication dynamics sensitive to the initial cell cycle stage [4]. VACV encodes a suite of proteins that are known to arrest and shift the cell cycle, leading to an accumulation of cells in the S/G2 phases [40]. This may reduce the extent to which cell cycle stage at the time of infection contributes to ongoing differences in infection outcomes.

Furthermore, our study uncovered a substantial shift in the between-cell variability of the onset of the early phase (Start Early) and the rate of virus production (Slope PR), which was associated with changing MOI. It might be reasonably assumed that the degree of variability is linked to the distribution of the number of infectious units a cell might encounter at different MOIs, a value which can be predicted by a Poisson distribution. For instance, at MOI 1, it is expected that 95% of cells receive 0–3 PFUs, whereas at MOI 100, this range extends to 81–120 PFUs—a 13-fold increase in the range of multiplicities leading to successful infection (Fig 6D). We observed that Start Early and Slope PR had contrasting relationships with these distributions: a broadening range of PFUs initiating an infection led to more consistent infection start times. Yet, it resulted in more variable virus production rates across the infected cell population (Fig 6E). This phenomenon was notably absent in PV infections, indicating a mechanism not shared by this virus.

The increasing consistency of infection start times suggests that VACV particles display cooperativity during the early stages of infection, whereby a greater number of infecting particles enhances the odds of success for each individual particle. While previous experiments with single VACV virions delivered to cells by atomic force microscopy combined with nanofluidics support the idea of cooperativity in the frequency of successful initiation of infection at lower MOIs [17], such cooperativity in other aspects of VACV infection has not been previously demonstrated. It is plausible that the reduction in infection start time variability seen in our data represents the stochastic nature of viral infection, wherein a greater number of infecting virions simply increases the probability of one optimally reaching the correct subcellular location and accumulating the necessary resources for early gene expression and productive replication. This phenomenon might also be a consequence of poxvirus-specific features, such as lateral bodies which deliver pre-packaged immunomodulatory proteins to the host cell shortly after entry [41–43], providing a mechanism by which individual particles could synergistically contribute to rapidly and reliably overcoming host defenses. The increasing variability in virus production rates may result from the nature of VACV replication centers, which increase in number with MOI, but appear to be limited above a certain threshold infectious dose [44]. Supporting this, we also observed a significant increase in the rate of early gene expression (Slope Early), which occurs within VACV cores and independent of a replication center [45], with increasing MOI.

Over the course of 36 HPI, the majority (62%) of cells infected at an MOI of 1 had lysed. The population of lytic and non-lytic infections possessed distinctive characteristics irrespective of whether lysis was defined at 24 or 36 HPI. Lytic infections represented a population with significantly accelerated progression from early to PR stage and displayed a truncated replicative phase resulting in reduced virus yield, despite replication proceeding at a similar rate to non-lytic infections once initiated. The role of lysis is not clear in the VACV infection cycle. There are two mature, infectious forms of the virus produced during infection, wrapped virus (WV) and mature virus (MV). WV are released through exocytosis, which occurs as early as 6–8 HPI [46]. The role of MV in cell-to-cell transmission is less clear as they do not traffic efficiently to the cell periphery [47] and are likely to be largely retained within the host cell until lysis, which is rare before 12 HPI [29]. In our data, cell lysis was observed in non-productive cells and was an earlier phenomenon than in productively infected cells. This indicates that the induction of programmed cell death functioned as a mechanism to curb virus production at the early-to-PR transition, and may also act on the population of virus-exposed cells before they progress to early gene expression. By inhibiting programmed cell death with the pan-caspase inhibitor Q-VD-OPh, the pool of cells susceptible to infection was expanded. The transition frequency from early to PR phase remained unaffected, collectively resulting in a greater number of productively infected cells. Surprisingly, this did not result in an overall increase in virus yield at the population level as there was a concomitant decrease in per cell yield (Figs 5A, 5C and S5). Blocking necroptosis with the addition of the RIPK1 inhibitor Necrostatin-1 did not enhance the effect of QVD on infection frequency, but did result in a small increase in the frequency of successful progression to productive replication. These results, for the first time, define multiple, opposing roles for caspases in VACV replication. While VACV encodes a multitude of caspase inhibitors [48], all known characterized functions are directed towards the inhibition of programmed cell death, including apoptosis and pyroptosis [25,49]. Inhibition of caspases by VACV gene functions is highly effective as caspase cleavage has only rarely been documented in wild-type infections. For example, cleavage of caspase-3 (VACV, Modified Vaccinia Ankara, MVA) and caspase-8 (MVA) has been detected at late stages of infection [18,30]. Pro-viral roles for caspase activity have been observed with other viruses, typically by leveraging caspase activity in order to activate viral proteins, or by exploiting caspase cleavage to dampen cellular immune defenses [50,51]. Whether the proviral role for caspases in VACV infection is actively regulated by viral factors is, at this stage, unknown.

Successful viral infection of a host cell represents the outcome of host and viral functions vying for control of the cellular environment. There is a growing consensus that how virus replication proceeds in the first cells during disease transmission will have an outsized impact on disease progression. For instance, transcriptomic and phenotypic analyses of cells in the nasal mucosa of SARS-CoV-2 infected patients revealed that severe disease progression was predicted by a failure of early infected cells to generate a robust antiviral innate immune response [52]. Bulk-cell analyses cannot address the mechanisms that contribute to this variability. By examining the single-cell infection dynamics of VACV, a large DNA virus with a multi-phase infection cycle, our results indicate that the pathway to productive infection must pass key bottlenecks. Apoptosis can eliminate infected cells before early viral gene expression, thus decreasing the pool of virus-producing cells. Furthermore, increasing the virus-to-cell ratio improves the likelihood and speed of early-stage cells progressing to PR stage, indicating that a higher virus-to-cell stoichiometry facilitates bypassing cellular defenses at the uninfected-to-early and early-to-PR transitions. Unlike the monophasic infection cycle of PV, where the dynamics of replication vary with MOI, productivity in VACV infection becomes host-limited once infection is established.

## Methods

### Cell culture

Cell lines were maintained in Dulbecco’s Modified Eagle’s Medium (DMEM, Gibco) supplemented with either 10% (HeLa: ATCC, CCL-2) or 5% (BS-C-1: ATCC, CCL-26) fetal bovine serum (FBS, Bovogen), 1% PSG (Gibco, 100 units/mL of penicillin, 100 μg/ml of streptomycin and 292 μg/ml L-glutamine), at 37 °C and 5% CO_2_ in a humidified incubator. Cells were routinely tested to confirm that they were free of Mycoplasma contamination (MycoAlert Mycoplasma Detection Kit, Lonza Bioscience).

### Virus stock preparation

Virus stocks were purified from the cytoplasmic fraction of infected cells by rate zonal sucrose gradient centrifugation (as in [53]). Titers of all virus stocks were determined by plaque assays on BS-C-1 cells. Purified VACV^Rep^ was serially diluted in 1mM Tris-HCl pH 9.0, wet mounted on a glass slide and imaged by widefield fluorescence microscopy. We were able to detect the fluorescence of the recombinant YFP-A3 protein, facilitating both confirmation of the appropriate dispersal of virus particles and direct counting. From this count (and PFU/mL assessed by plaque assay) a plaque-to-PFU ratio of 15.4 was calculated (S6 Fig).

### VACV infections

Except where otherwise specified, all infections were performed as follows. Growth medium was aspirated from cells, followed by a single wash with PBS. Virus, at a predefined MOI, was then diluted in DMEM not supplemented with FBS (Serum-Free Medium; SFM) and overlaid onto cells for 1 hr at 37°C in a 5% CO_2_ atmosphere. After this incubation period virus inocula were aspirated, cells were washed once with warm PBS, and overlaid with the appropriate fresh growth medium.

### Construction and characterization of VACV^Rep^

VACV^Rep^ was generated by recombination of VACV WR pE/L-mCh [18] and VACV WR YFP-A3 [19] in co-infected BS-C-1 cells. After 48 h, cells were harvested and lysed by freeze-thawing. The resulting lysates were serially diluted and used to infect confluent monolayers of BS-C-1 cells as detailed above. Plaques expressing both pE/L-mCh and YFP-A3 (as detected by fluorescence) were selected, isolated, and subjected to 6 rounds of plaque-purification on monolayers of BS-C-1 cell.

To assess the attenuation of VACV^Rep^, confluent BS-C-1 monolayers were infected with ∼50 PFU and plaques were analyzed as detailed above (S7 Fig).

To confirm that YFP fluorescence required PR gene expression HeLa cells were infected with VACV^Rep^ at an MOI of 5 and treated with either vehicle (DMSO) or araC (Sigma-Aldrich), and fixed in 4% paraformaldehyde (PFA) at 8 HPI and 24 HPI. Cells were imaged on a widefield fluorescence microscope (Nikon Ti-E) and fluorescence of both mCherry and YFP were quantified at each time point (S8 Fig).

To validate that YFP fluorescence is a reliable marker for the production of infectious progeny virus, HeLa cells were seeded into duplicate 24-well tissue culture plates at 70% confluency, infected with VACV^Rep^ at an MOI of 5 and treated with either 10 μM araC (Sigma-Aldrich) or an equivalent volume of vehicle (DMSO). The fluorescence of YFP and mCherry was quantitatively measured across the entire well surface using contiguous field imaging at 0, 6, 8, 10, 12, and 24 HPI, following the replacement of the viral inoculum with fresh medium. Cells and media from the corresponding wells on the second plate were harvested at the same time points, and the quantity of progeny virus assessed by plaque assay (S1 Fig). For plaque quantification, cells were fixed in 4% PFA at 48 HPI, and mCherry signal was imaged using a Bio-Rad ChemiDoc MP imaging system. Plaque counts were performed using the Fiji software ([54] v1.53t).

### Plaque assays

BS-C-1 cells were seeded into 6-well tissue culture plates and grown to confluence. Virus strains were diluted in SFM to the appropriate concentration and added to cells. Cells were incubated at 37°C, 5% CO_2_ for 1 hr, washed with PBS, then overlaid with 1x Minimal Essential Medium (MEM, Gibco) containing 1.5% carboxy-methyl cellulose (CMC) and supplemented with 1% PSG (Gibco, 100 units/mL of penicillin, 100 μg/ml of streptomycin and 292 μg/ml L-glutamine) and 5% FBS for standard plaque assays, or 1x Minimal Essential Medium (MEM, Gibco) containing 0.9% (w/v) Ultrapure Agarose (Invitrogen) and supplemented with 1% PSG (Gibco, 100 units/mL of penicillin, 100 μg/ml of streptomycin and 292 μg/ml L-glutamine) and 5% FBS for plaque isolation. VACV plaques were grown for 2–3 days, then either stained with crystal violet or imaged by widefield fluorescence microscopy as indicated. For crystal violet stained monolayers plaques were imaged using a LI-COR Odyssey CLx Imager on white light mode. Plaque size was quantified using Fiji ([55] v1.53t).

### One-step growth assay

HeLa cells were infected with VACV^Rep^ at an MOI of 5 as detailed above. Cells and supernatant were harvested at the indicated times, freeze thawed 3 times in liquid nitrogen to release virus, and titered by plaque assay on confluent BS-C-1 cells.

### Live fluorescence microscopy of VACV infected cells

To minimize extraneous sources of variability, we implemented a number of experimental measures. Virus was purified by sucrose gradient isopycnic centrifugation (S9 Fig), subjected to mild sonication for improved virus particle dispersion, and virus entry into cells was synchronized by binding at 4°C, a temperature which permits binding but not fusion of VACV particles [56,57]. To restrict virus transmission between infected cells, we utilized an overlay containing CMC [58].

To image live VACV^Rep^ infected cells HeLa cells were resuspended, passed through a cell strainer (Corning) to eliminate aggregates, and seeded into a poly-L-lysine (Sigma-Aldrich) coated 12-well glass-bottom tissue culture plate (Cellvis) at 2.5% confluency, corresponding to approximately 12,500 cells per well. Post-seeding, cells were permitted an 8 hr adhesion period at 37°C and 5% CO2. Following this, they were transferred to 4°C for 15 minutes, washed with 4°C PBS, and overlaid with virus diluted to the appropriate MOI in 4°C serum-free medium (SFM) containing 10mM HEPES. Cells were then incubated with virus at 4°C (a temperature which permits binding but not entry of VACV) for 1 hr.

Post-incubation cells were washed with room-temperature PBS and overlaid with imaging medium (1x Minimum Essential Medium (MEM), 1.5% CMC, 10% FBS). Imaging was carried out using a Nikon Eclipse Ti-E Microscope outfitted with a motorized stage, an iXon Ultra 888 EMCCD (Andor) camera and an environmental chamber maintained at 37°C and 5% CO_2_ (Okolab, Cage incubator).

Images were obtained at 5–10 minute intervals over a 24–36 hr period, in Phase Contrast, red (TxRED Semrock, 300ms exposure time) and green (FITC Semrock, 300ms exposure time) channels with a x10 objective. Image acquisition was controlled using NIS-Elements Microscope Imaging Software AR (Nikon, v4.51.01).

To confirm the suitability of our system for long term live imaging we performed a mock infection as above, with the addition of 2.5 μg/ml Propidium Iodide to the imaging medium, and imaged cells for 48 hrs to assess their growth rate and viability (S10 Fig).

### Programmed cell death inhibition in infected VACV^Rep^ cells

For single-cell imaging experiments cells were infected as previously described, with the inclusion of either 50 μM Q-VD-OPh (QVD, MedChemExpress), or 50 μM QVD and 40 μM Necrostatin-1 (Nec-1, MedChemExpress), or an equivalent volume of DMSO (for vehicle controls). QVD or QVD/Nec-1 were present during both the 1 hr binding phase at 4°C and in the imaging medium overlay.

Plaque assays with QVD were performed as detailed above, but with the addition of either 50 μM QVD or an equivalent volume of DMSO (for vehicle controls) in the plaque overlay medium.

### Data analysis

Single cell experiment images captured in the fluorescence channels were corrected to account for background noise and photobleaching over time using the Background Subtraction and Image Correction (BaSiC) tool ([59], available as a Fiji software plugin). Phase contrast images were processed for pixel classification by a Random Forest classifier, which was trained on a subset of the data to automate cell identification (Ilastik v1.4.0rc2 [60]). The output probability maps were exported and utilized to generate a mask for fluorescence channel images. This facilitated the segmentation, object identification, declumping, tracking (by the Linear Assignment Problem method), and fluorescence quantification of individual cells over time (CellProfiler v4.2.4 [61]). Approximately 30% of the imaged cells were unable to be analyzed due to exiting the field of view, merging with other cells, or dividing. As a result of this phase contrast segmentation and tracking approach our pipeline was also able to collect data on a number of morphological and behavioral cellular characteristics, including motility over time, size, and various shape descriptors including compactness (the quotient of the mean squared distance of the object’s pixels from the centroid and the area, a measure of roundness).

Data management and modeling were performed in R statistical software. Single cell sigmoidal curve fitting was accomplished with a custom modification of the Sigmoidal curve fitting for Cell Growth Analysis in R (SICEGAR) package [62].

Single-cell infection dynamics parameters were defined as follows:

- Start Early: The time point at which a cell’s mCherry signal integrated density exceeds 1.5 times the mCherry cutoff score (calculated from the mean and standard deviation of the mCherry integrated density of uninfected control cells).
- Slope Early: The slope of the fitted curve at Midpoint Early.
- Start PR: The time point at which a cell’s YFP signal integrated density exceeds 1.5 times the YFP cutoff score (calculated from the mean and standard deviation of the YFP integrated density of uninfected control cells).
- Midpoint PR: The time point at which the slope of the fitted curve is maximal and the intensity equal to half of Max PR.
- Slope PR: The slope of the fitted curve at Midpoint PR.
- Period PR: The time difference between when the tangent line passing through the midpoint with slope equal to Slope PR intersects with the y-axis, and when it intersects with the horizontal line defined by Max PR.
- Max PR: The maximum intensity of the fitted curve.

With the exception of Start Early and Start PR(for which we have used modified definitions in this study) complete mathematical details of all parameters can be found in the SICEGAR package documentation. The values of both Start Early and Start PR should be considered in the context of the maturation times of their associated fluorophores (mCherry: ∼15 min [63] and YFP: ∼ 5 min [64] at 37°C).

### Statistical analysis

All statistical analyses were performed using R statistical software ([65], v4.3.1). Statistical tests (rstatix v0.7.2). Sample size, number of replicates, and the statistical tests performed are indicated in the appropriate figure legends and provided in full for all single cell infection dynamics parameters in S5 Table. Annotations of significance indicate the following p-values: * p<0.05, ** p<0.01, ***, p<0.001, **** p<0.0001.

## Supporting information

S1 Video VACV^Rep^ enables visualization of early and PR gene expression phases in infected cells. **Time**-lapse live-cell fluorescence microscopy of VACV^Rep^ infected HeLa cells. Images were acquired at 10 minute intervals with a 40x objective. Scale bar indicates 20 μm.
(MP4)

S1 Fig Correlation between YFP-A3 fluorescence intensity and progeny infectious virus production. A, Sequential fluorescence images of representative wells depicting the time-dependent increase in progeny virus produced in HeLa cells post-infection with VACV^Rep^, monitored at 0, 6, 8, 10, 12, and 24 HPI. Cells were treated with (+) or without (-) 10 μM araC to inhibit viral DNA replication. B, Linear regression correlating mean fluorescence intensity (NFI) with the titre (PFU/mL) of progeny virus produced, as assessed by plaque assay. The fluorescence intensity across the entire well was quantified. C, Normalized (to the maximum value observed for each parameter by min-max normalization) mean values of fluorescence intensity (for mCherry and YFP-A3) and virus titer at 0 and 24 HPI, with and without araC treatment (n = 2 wells). Error bars represent SD.
(EPS)

S2 Fig Correlations between morphological characteristics and PR infection parameters at the single-cell level. Parameters extracted from models fitted to single-cell infection timeseries PR fluorescence data (MOI 1; n = 88 cells, MOI 10; n = 232 cells, MOI 50; n = 205 cells, MOI 100; n = 200 cells) were analyzed via pairwise correlations (Pearson’s correlation coefficient) with morphological parameters mean cell area (mean area), total distance moved over the duration of the experiment (motility), and shape (compactness, an indicator of roundness in which one indicates perfect roundness and values greater than one indicate progressively less round shapes) with all PR single cell infection parameters. This indicates the presence or absence of correlation between virus production and readily observable morphological attributes at the single-cell level.
(EPS)

S3 Fig Single-cell infection parameters for non-lytic and lytic cells when determined at different times points. Parameters extracted from models fitted to VACV^Rep^ single-cell infection curves at an MOI of 1 were evaluated by their density distributions (Student’s T-test) for both lytic and non-lytic cell populations. The dataset was restricted to 24 HPI.
(EPS)

S4 Fig The impact of pan-caspase inhibition on pairwise relationships of single-cell infection dynamics parameters. Parameters extracted from models fitted to single-cell infection fluorescence data (Vehicle; n = 76 cells, Vehicle non-lytic; n = 66 cells, QVD; n = 112 cells) were analyzed via pairwise correlations (Pearson’s correlation coefficient).
(EPS)

S5 Fig The impact of pan-caspase inhibition on VACV^Rep^ replication dynamics at the population level. HeLa cells were seeded into a 12-well tissue culture plate, grown to approximately 80% confluency, and infected with VACV^Rep^ at an MOI of 5, with or without the addition of 50 μM QVD to the rescue medium. Cells and supernatant were collected at 6, 8, 24, and 48 hrs and titers were determined by plaque assay on BS-C-1 cells. Datapoints show the summed titer of supernatant and cell fractions, and are the mean of three biological replicates. Error bars indicate SEM. Significance levels shown represent the results of unpaired Student’s t-tests.
(EPS)

S6 Fig Direct counting of VACV^Rep^ particles. VACV^Rep^ purified by rate zonal sucrose gradient centrifugation was sonicated briefly then serially diluted in 1mM Tris-HCl pH 9.0, wet mounted onto a glass slide, and imaged by widefield fluorescence microscopy (FITC filter; 495 nm excitation, 519 nm emission). Images were analyzed in Fiji 2.9.0. Arrows indicate single VACV particles.
(EPS)

S7 Fig Characterization of VACV^Rep^ by plaque assay. Confluent BS-C-1 cells were infected with serial dilutions of either VACV^Rep^, VACV WR, or VACV WR pE/L-mCherry and overlaid with rescue medium containing 50% carboxymethylcellulose (CMC). AT 48 hrs post infection the overlay was removed and the cells were stained with crystal violet and imaged. Data represents the mean of 3 biological replicates. Error bars indicate SEM. Statistical analysis was performed by ANOVA followed by TukeyHSD post-hoc testing.
(EPS)

S8 Fig Expression of YFP-A3 requires progression to PR phase gene expression. HeLa cells were seeded onto poly-L-lysine coated coverslips at low density and infected with VACV^Rep^ at an MOI of 5, with or without the addition of 10 μM araC to the rescue medium. Cells were fixed with PFA at 8 and 24 HPI and mounted on glass slides for imaging. 40 randomly selected cells were measured for mCherry and YFP fluorescence for each condition. Error bars indicate SEM. Statistical comparisons were performed using unpaired Student’s T-tests.
(EPS)

S9 Fig Crude versus highly purified VACV preparations. Rate zonal centrifugation of VACV eliminates large virus laden debris from the preparation. Arrows indicate virus laden aggregates of debris (**A**) and single virus particles adhered to susceptible cells (**B**), respectively.
(EPS)

S10 Fig Long-term viability of cells under single-cell imaging conditions. HeLa cells were mock infected and placed into our live imaging system with the addition of propidium iodide (PI) to imaging medium. Confluency (Average % of all fields of view covered by cells) and viability (% of cells PI+) was monitored over 40 hrs. Confluency values were normalized to starting cell density.
(EPS)

S1 Table Counts of fitted sigmoidal curves for all single-cell experiment data. Counts indicate the number of single-cell infection curves that were fitted by the indicated model. Sigmoidal corresponds to a persistent infection. Double-sigmoidal corresponds to a lytic infection. Ambiguous indicates that neither a sigmoidal nor double-sigmoidal curve could be fitted. Visual inspection suggests that the majority of ambiguous fits result from cells expressing extremely low levels of the indicated reporter at a slow rate.
(DOCX)

S2 Table Variability of single-cell infection parameters at different MOIs. Gini coefficients were calculated for each parameter at each MOI. A lower Gini coefficient indicates a more even distribution of values of the indicated parameter among all infections, while a higher Gini coefficient indicates the opposite.
(DOCX)

S3 Table The impact of MOI on the frequency of lysis of productive and non-productive infections. Counts of lytic and non-lytic cells for productive (PR NFI positive) and non-productive (PR NFI negative) infections.
(DOCX)

S4 Table The impact of pan-caspase inhibition on the frequency of uninfected cell death. Counts of live and dead uninfected (Early NFI negative) cells with and without the presence of the pan-caspase inhibitor QVD. Dead cells were identified by their characteristic lack of motility and morphological staticity.
(DOCX)

S5 Table Statistical test results for pairwise comparisons of single-cell infection dynamics parameters. Adjusted p-values of t-tests performed on single cell infection data. All p-values shown were calculated by pairwise t-tests and adjusted for multiple comparisons with Bonferroni’s correction. Relates to Figs 4 and 5.
(DOCX)
