# Supplementary material for: Single-cell analysis of VACV infection reveals pathogen-driven timing of early and late phases and host-limited dynamics of virus production
Source: PLoS Pathog. 2024 Aug 2;20(8):e1012423. doi: 10.1371/journal.ppat.1012423 (PMC11347022; doi:10.1371/journal.ppat.1012423)
Supplement: S1 Table — Counts indicate the number of single-cell infection curves that were fitted by the indicated model. Sigmoidal corresponds to a persistent infection. Double-sigmoidal corresponds to a lytic infection. Ambiguous indicates that neither a sigmoidal nor double-sigmoidal curve could be fitted. Visual inspection suggests that the majority of ambiguous fits result from cells expressing extremely low levels of the indicated reporter at a slow rate. (DOCX) [file ppat.1012423.s012.docx]

| **MOI** | **Reporter** | **Sigmoidal** | **Double-sigmoidal** | **No signal** | **Ambiguous** |
| --- | --- | --- | --- | --- | --- |
| 1 | Early | 32 | 56 | N/A | 3 |
| 10 | Early | 83 | 173 | N/A | 1 |
| 50 | Early | 77 | 157 | N/A | 4 |
| 100 | Early | 53 | 189 | N/A | 4 |
| 1 | PR | 37 | 3 | 50 | 1 |
| 10 | PR | 209 | 38 | 10 | 0 |
| 50 | PR | 207 | 29 | 2 | 0 |
| 100 | PR | 214 | 30 | 2 | 0 |
| **Treatment** | **Reporter** | **Sigmoidal** | **Double-sigmoidal** | **No signal** | **Ambiguous** |
| Vehicle | Early | 84 | 34 | N/A | 2 |
| QVD | Early | 132 | 3 | N/A | 1 |
| Vehicle | PR | 85 | 0 | 44 | 9 |
| QVD | PR | 86 | 1 | 58 | 4 |
