# Supplementary material for: Single-cell analysis of VACV infection reveals pathogen-driven timing of early and late phases and host-limited dynamics of virus production
Source: PLoS Pathog. 2024 Aug 2;20(8):e1012423. doi: 10.1371/journal.ppat.1012423 (PMC11347022; doi:10.1371/journal.ppat.1012423)
Supplement: S2 Table — Gini coefficients were calculated for each parameter at each MOI. A lower Gini coefficient indicates a more even distribution of values of the indicated parameter among all infections, while a higher Gini coefficient indicates the opposite. (DOCX) [file ppat.1012423.s013.docx]

| MOI | Start Early | Start PR | Midpoint PR | Slope PR | Period PR | Max PR |
| --- | --- | --- | --- | --- | --- | --- |
| 1 | 0.259 | 0.193 | 0.179 | 0.227 | 0.299 | 0.382 |
| 10 | 0.155 | 0.129 | 0.176 | 0.265 | 0.343 | 0.360 |
| 50 | 0.116 | 0.125 | 0.165 | 0.275 | 0.313 | 0.360 |
| 100 | 0.110 | 0.158 | 0.172 | 0.283 | 0.311 | 0.373 |
