# Supplementary material for: Single-cell analysis of VACV infection reveals pathogen-driven timing of early and late phases and host-limited dynamics of virus production
Source: PLoS Pathog. 2024 Aug 2;20(8):e1012423. doi: 10.1371/journal.ppat.1012423 (PMC11347022; doi:10.1371/journal.ppat.1012423)
Supplement: S3 Table — Counts of lytic and non-lytic cells for productive (PR NFI positive) and non-productive (PR NFI negative) infections. (DOCX) [file ppat.1012423.s014.docx]

| MOI | Non-productive Lytic | Non-productive non-lytic | Productive Lytic | Productive non-lytic |
| --- | --- | --- | --- | --- |
| 1 | 28 | 19 | 28 | 13 |
| 10 | 7 | 1 | 166 | 82 |
| 50 | 1 | 0 | 156 | 77 |
| 100 | 1 | 0 | 188 | 53 |
