# Supplementary material for: Single-cell analysis of VACV infection reveals pathogen-driven timing of early and late phases and host-limited dynamics of virus production
Source: PLoS Pathog. 2024 Aug 2;20(8):e1012423. doi: 10.1371/journal.ppat.1012423 (PMC11347022; doi:10.1371/journal.ppat.1012423)
Supplement: S4 Table — Counts of live and dead uninfected (Early NFI negative) cells with and without the presence of the pan-caspase inhibitor QVD. Dead cells were identified by their characteristic lack of motility and morphological staticity. (DOCX) [file ppat.1012423.s015.docx]

| Condition | Well ID | Live uninfected | Dead uninfected | Proportion dead |
| --- | --- | --- | --- | --- |
| Control | 5 | 24 | 12 | 0.33 |
| Control | 9 | 31 | 8 | 0.205 |
| QVD | 2 | 7 | 0 | 0 |
| QVD | 6 | 14 | 0 | 0 |
| QVD | 10 | 4 | 2 | 0.33 |
