# Supplementary material for: Single-cell analysis of VACV infection reveals pathogen-driven timing of early and late phases and host-limited dynamics of virus production
Source: PLoS Pathog. 2024 Aug 2;20(8):e1012423. doi: 10.1371/journal.ppat.1012423 (PMC11347022; doi:10.1371/journal.ppat.1012423)
Supplement: S5 Table — Adjusted p-values of t-tests performed on single cell infection data. All p-values shown were calculated by pairwise t-tests and adjusted for multiple comparisons with Bonferroni’s correction. Relates to Figs 4 and 5. (DOCX) [file ppat.1012423.s016.docx]

| Start Early |  | **MOI 1** | **MOI 10** | **MOI 50** | **MOI 100** | **Control** | **QVD** | **Sample size** |
| --- | --- | --- | --- | --- | --- | --- | --- | --- |
|  | **MOI 1** | 1 | 4.09E-90 | 3.30E-121 | 2.21E-126 |  |  | 88 |
|  | **MOI 10** | 4.09E-90 | 1 | 8.53E-12 | 4.79E-15 |  |  | 232 |
|  | **MOI 50** | 3.30E-121 | 8.53E-12 | 1 | 1 |  |  | 205 |
|  | **MOI 100** | 2.21E-126 | 4.79E-15 | 1 | 1 |  |  | 200 |
|  | **Control** |  |  |  |  | 1 |  | 76 |
|  | **QVD** |  |  |  |  |  | 1 | 81 |
|  | **Sample size** | 88 | 232 | 205 | 200 | 76 | 81 |  |
| Start PR |  | **MOI 1** | **MOI 10** | **MOI 50** | **MOI 100** | **Control** | **QVD** | **Sample size** |
|  | **MOI 1** | 1 | 1.03E-89 | 1.13E-130 | 3.63E-142 |  |  | 88 |
|  | **MOI 10** | 1.03E-89 | 1 | 1.41E-32 | 7.94E-50 |  |  | 232 |
|  | **MOI 50** | 1.13E-130 | 1.41E-32 | 1 | 0.004975 |  |  | 205 |
|  | **MOI 100** | 3.63E-142 | 7.94E-50 | 0.004975 | 1 |  |  | 200 |
|  | **Control** |  |  |  |  | 1 |  | 76 |
|  | **QVD** |  |  |  |  |  | 1 | 81 |
|  | **Sample size** | 88 | 232 | 205 | 200 | 76 | 81 |  |
| Midpoint PR |  | **MOI 1** | **MOI 10** | **MOI 50** | **MOI 100** | **Control** | **QVD** | **Sample size** |
|  | **MOI 1** | 1 | 1.62E-36 | 2.45E-53 | 2.01E-58 |  |  | 88 |
|  | **MOI 10** | 1.62E-36 | 1 | 2.74E-09 | 1.62E-14 |  |  | 232 |
|  | **MOI 50** | 2.45E-53 | 2.74E-09 | 1 | 0.557446 |  |  | 205 |
|  | **MOI 100** | 2.01E-58 | 1.62E-14 | 0.557446 | 1 |  |  | 200 |
|  | **Control** |  |  |  |  | 1 | 2.279080e-01 | 76 |
|  | **QVD** |  |  |  |  | 2.279080e-01 | 1 | 81 |
|  | **Sample size** | 88 | 232 | 205 | 200 | 76 | 81 |  |
| Slope PR |  | **MOI 1** | **MOI 10** | **MOI 50** | **MOI 100** | **Control** | **QVD** | **Sample size** |
|  | **MOI 1** | 1 | 1 | 0.067401 | 0.048759 |  |  | 88 |
|  | **MOI 10** | 1 | 1 | 0.139144 | 0.076888 |  |  | 232 |
|  | **MOI 50** | 0.067401 | 0.139144 | 1 | 1 |  |  | 205 |
|  | **MOI 100** | 0.048759 | 0.076888 | 1 | 1 |  |  | 200 |
|  | **Control** |  |  |  |  | 1 | 6.149757e-01 | 76 |
|  | **QVD** |  |  |  |  | 6.149757e-01 | 1 | 81 |
|  | **Sample size** | 88 | 232 | 205 | 200 | 76 | 81 |  |
| Period PR |  | **MOI 1** | **MOI 10** | **MOI 50** | **MOI 100** | **Control** | **QVD** | **Sample size** |
|  | **MOI 1** | 1 | 1 | 1 | 1 |  |  | 88 |
|  | **MOI 10** | 1 | 1 | 1 | 1 |  |  | 232 |
|  | **MOI 50** | 1 | 1 | 1 | 1 |  |  | 205 |
|  | **MOI 100** | 1 | 1 | 1 | 1 |  |  | 200 |
|  | **Control** |  |  |  |  | 1 | 4.070054e-01 | 76 |
|  | **QVD** |  |  |  |  | 4.070054e-01 | 1 | 81 |
|  | **Sample size** | 88 | 232 | 205 | 200 | 76 | 81 |  |
| Max PR |  | **MOI 1** | **MOI 10** | **MOI 50** | **MOI 100** | **Control** | **QVD** | **Sample size** |
|  | **MOI 1** | 1 | 1 | 0.4097 | 0.572605 |  |  | 88 |
|  | **MOI 10** | 1 | 1 | 0.112575 | 0.231757 |  |  | 232 |
|  | **MOI 50** | 0.4097 | 0.112575 | 1 | 1 |  |  | 205 |
|  | **MOI 100** | 0.572605 | 0.231757 | 1 | 1 |  |  | 200 |
|  | **Control** |  |  |  |  | 1 | 1.317031e-01 | 76 |
|  | **QVD** |  |  |  |  | 1.317031e-01 | 1 | 81 |
|  | **Sample size** | 88 | 232 | 205 | 200 | 76 | 81 |  |
